# Cultural adaptation into Spanish of the generalized anxiety disorder-7 (GAD-7) scale as a screening tool

**DOI:** 10.1186/1477-7525-8-8

**Published:** 2010-01-20

**Authors:** Javier García-Campayo, Enric Zamorano, Miguel A Ruiz, Antonio Pardo, María Pérez-Páramo, Vanessa López-Gómez, Olga Freire, Javier Rejas

**Affiliations:** 1 Department of Psychiatry, Hospital Universitario Miguel Servet, Zaragoza, Spain; 2 ISCIII- REDIAPP, Red de Investigación en Actividades Preventivas y Promoción de la Salud, Zaragoza, Spain; 3 Sant Antoni de Vilamajor Primary Care Health Center, ABS Alt Mogent, Barcelona, Spain; 4 Department of Methodology, School of Psychology, Universidad Autónoma de Madrid, Madrid, Spain; 5 EACCOS Research Group, Madrid, Spain; 6 Neuroscience Department, Medical Unit, Pfizer Spain, Alcobendas (Madrid), Spain; 7 Health Outcomes Research Department, Medical Unit, Pfizer Spain, Alcobendas (Madrid), Spain

## Abstract

**Background:**

Generalized anxiety disorder (GAD) is a prevalent mental health condition which is underestimated worldwide. This study carried out the cultural adaptation into Spanish of the 7-item self-administered GAD-7 scale, which is used to identify probable patients with GAD.

**Methods:**

The adaptation was performed by an expert panel using a conceptual equivalence process, including forward and backward translations in duplicate. Content validity was assessed by interrater agreement. Criteria validity was explored using ROC curve analysis, and sensitivity, specificity, predictive positive value and negative value for different cut-off values were determined. Concurrent validity was also explored using the HAM-A, HADS, and WHO-DAS-II scales.

**Results:**

The study sample consisted of 212 subjects (106 patients with GAD) with a mean age of 50.38 years (SD = 16.76). Average completion time was 2'30''. No items of the scale were left blank. Floor and ceiling effects were negligible. No patients with GAD had to be assisted to fill in the questionnaire. The scale was shown to be one-dimensional through factor analysis (explained variance = 72%). A cut-off point of 10 showed adequate values of sensitivity (86.8%) and specificity (93.4%), with AUC being statistically significant [AUC = 0.957-0.985); p < 0.001]. The scale significantly correlated with HAM-A (0.852, p < 0.001), HADS (anxiety domain, 0.903, p < 0.001), and WHO-DAS II (0.696, p > 0.001).

**Limitations:**

Elderly people, particularly those very old, may need some help to complete the scale.

**Conclusion:**

After the cultural adaptation process, a Spanish version of the GAD-7 scale was obtained. The validity of its content and the relevance and adequacy of items in the Spanish cultural context were confirmed.

## Introduction

Anxiety is the manifestation of an emotion where the individual feels and describes him/herself as restless, nervous, tense, afraid or excessively worried about specific, or perhaps undefined, issues [1]. Both the DSM-IV and the ICD-10 clearly distinguish between generalized anxiety disorders (GAD) and other anxiety and depression conditions [2]. While the exact cause of GAD cannot be specified, there are population groups at greater risk of suffering it [3-5]. As other anxiety disorders, GAD is associated to many diseases, both psychiatric and organic [6]. The impact of GAD on health-related quality of life is even greater than that observed in major depression [7], and changes cannot be totally accounted for by comorbid diseases.

Anxiety disorders are the most common mental disorders in Europe, with a one-year prevalence of 12% in the European adult population [5], 12-month prevalence in the adult population of 2% to 3%, and a lifetime prevalence of 5% [5]. According to data from the European Study of the Epidemiology of Mental Disorders (ESEMeD), conducted in six European countries including Spain, the last-year prevalence and the lifetime prevalence of anxiety disorders are 6% and 13.6% respectively [8]. Lifetime prevalence in individuals specifically diagnosed with GAD is estimated at 5.1% (DSM-IV), and at 6.5% according to the classification criteria used in Europe (ICD-10) [1,9,10]. It is thought that more than half the patients with anxiety disorders attend primary care centers. Since approximately 8% of these patients are diagnosed with GAD, it follows that this is the most prevalent anxiety disorder [11,12]. GAD prevalence in primary care studies carried out in Spain ranges from 4.5% and 7.9% [1,13].

Nosological changes have led the scientific community to develop specific, psychometrically sound measurement tools able to identify and quantify the intensity of GAD according to its current conception, and to assess the efficacy of psychosocial and psychopharmacological interventions in these patients. Many health questionnaire-based instruments allowing clinicians to approach GAD at any healthcare level are currently available. These tools, or health measurement scales, may be used for several purposes. They allow for objective quantification of the presence and intensity of the main symptoms of GAD, or may help identify probable cases of GAD. Scales validated in our culture and routinely used include the Hamilton Anxiety Scale (HAM-A) [14], Hospital Anxiety and Depression Scale (HADS) [15], Covi Anxiety Scale [16], Clinical Anxiety Scale (CAS) [17], and State-Trait Anxiety Inventory (STAI) [18], among others. Regardless of whether they are clinician-administered structured interviews or self-administered questionnaires to be completed by the patient, all these scales assess the presence of anxiety symptoms and their intensity, but are not suitable for early detection or identification of probable GAD cases by patient self-administration unless clinically supervised.

Different scales have been developed in recent years in an attempt to measure the construct of pathological worry (the core symptom of GAD), or simply worry, as an approach to GAD detection, even in the primary care setting. Only two instruments, the Anxiety Screening Questionnaire (ASQ-15) [19] and the screening scale for DSM-IV General Anxiety Disorder of Carroll and Davidson [20] (the latter based on DSM-IV criteria), have been designed to evaluate each and every manifestation currently defining GAD. The ASQ-15 allows for detecting GAD according to DSM-IV and ICD-10 criteria, as well as other anxiety symptoms, but no version adapted to and validated for our culture is available. On the other hand, the 12-item Carroll and Davidson scale, of which a linguistically and psychometrically validated Spanish version is available [21], allows for identifying GAD patients according to DSM-IV criteria only.

Recently, a simple 7-item tool, also based on DSM-IV criteria, has been validated to identify probable cases of GAD: the GAD-7 scale [22]. This patient- and clinician-friendly instrument has shown excellent properties to identify patients with probable GAD, is easy to administer, and does not represent an overburden for patients or clinicians. In addition, its briefness makes it suitable for use in epidemiological studies and for potential use in surveys with remote administration of health questionnaires. No version adapted to our culture is currently available.

The objective of this study was to create a version of the GAD-7 questionnaire culturally adapted to the Spanish language, and to assess the psychometric properties of the adapted version in terms of reliability and validity.

## Materials and methods

The GAD-7 questionnaire is a one-dimensional self-administered scale designed to assess the presence of the symptoms of Generalized Anxiety Disorder (GAD), as listed in the DSM-IV. The contents of the questionnaire were selected by the original authors from a larger list of symptoms. Since the Spanish version has inherited these contents, its content validity is justified by the original version. Since the objective was to obtain an instrument as similar to the original as possible, extraction of additional contents was considered inappropriate. The methodology currently recommended for adaptation of psychometric instruments was used [23,24]; assumptions of the Classical Test Theory were also used [25]. The total GAD-7 score is calculated by simple addition of the answers to each item. Scores for all 7 items range from 0 (Not at all) and 3 (Nearly every day). Therefore, the total score ranges from 0 and 21. According to the original authors [22], the total score may be categorized into four severity groups: minimal (0-4), mild (5-9), moderate (10-14) and serious (14-20)[22].

The process of cultural adaptation of the questionnaire started with duplicate translations of the English original into Spanish by two separate English-speaking native translators. Both translations were reviewed by an Expert Panel consisting of 4 clinicians (including a psychiatrist), 1 expert in clinical research, and 2 methodologists specialized in measurement tasks. Both translations were then merged into a single reconciled version, which was subject to a content validity process by interrater agreement estimation. A panel of 8 specialists in psychiatric disorders was selected for this purpose. These specialists independently assessed whether each item did or did not properly measure GAD (objective concept) and whether it could or could not measure depression (distractor concept). The index of item-objective congruence was computed from the specialists' ratings [26]. This index has a value of 1 in the event of perfect congruence in assigning the item to one domain, and a valued of -1 when such congruence is lacking.

After content validity assessment, a pilot test was conducted on a reduced sample of patients and control subjects to assess understandability and feasibility of the translation in real subjects. Completion time was also estimated. As the questionnaire will be used in the future to identify possible GAD cases, a subsample of healthy subjects was included to further assess understandability. The reconciled version was administered to the pilot sample together with a brief additional questionnaire to ascertain the help needed to complete the questionnaire, the difficulties encountered, and sociodemographic variables. In view of the results obtained in the pilot test, the questionnaire header was modified to emphasize the frequency of symptom onset; the anchors of the response categories were also modified. The final version was translated back into English by two separate translators and sent to the original authors for conceptual equivalence assessment.

Once piloted, the final version was included in a Case Report Form (CRF) for administration to the scaling and validation sample subjects in order to determine the psychometric properties of the final version. The CRF included information on disease diagnosis, sociodemographic variables, disease treatment variables and concomitant diseases, several concurrent questionnaires of patient-reported measures, and information on the number of visits to primary care and specialist physicians.

### Study design

This was a multicentre, cross-sectional study to test the feasibility, reliability, and validity of a new health technology conducted under standard clinical practice conditions. All patients were required to give their informed consent to participate in the study and have their data analyzed. The study protocol was approved by the Research Committee of the Universidad Autónoma de Madrid.

Two measurements were performed: (1) *Test*: at recruitment, the GAD questionnaire was administered to the sample of patients and controls together with three other instruments: the Hamilton Anxiety Scale (HAM-A) [14], the Hospital Anxiety and Depression Scale (HADS) [15], and the WHO-DAS II disability questionnaire [27,28], all of them in their Spanish versions (see below). Sociodemographic and medical history data were recorded at this visit. (2) *Retest*: one week later, the GAD-7 scale was again administered to a patient subsample; the retest information was used to study the stability of questionnaire measurements over time. The latter was ensured by administering the HADS concomitantly with GAD-7 one week after the first administration.

### Samples

The pilot sample consisted of 16 subjects selected from those attending the practices of two of the participating clinical investigators. Eight patients regularly attending the practices with a prior diagnosis of clinical GAD were randomly selected. A sample of 8 GAD-free subjects, sex- and age-matched to patients with GAD, was also selected.

To obtain the validation and scaling sample, each investigator had to recruit from 12 to 24 patients, half of them diagnosed with GAD and the other half as sex- and age-matched (± 5 years) controls. Patients were randomly selected by 14 investigators (family physicians in urban areas in the provinces of Madrid, Zaragoza, and Barcelona) among those attending their practices. Inclusion criteria were as follows: patients of both sexes over 18 years of age; able to speak and understand Spanish; with a known diagnosis of GAD (for the GAD group of patients, diagnosis performed under standard medical practice conditions according to the DSM-IV-TR classification was required) or no diagnosis of any anxiety disorder for control subjects (HAM-A < 10); under no anxiolytic treatment, or receiving anxiolytic therapy but with presence of anxiety symptoms (score ≥ 16 points in the HAM-A anxiety scale) for the GAD group. Exclusion criteria included: patients or subjects who were in a health state which did not allow for scale self-administration in the investigator's judgment; patients unable to understand or answer the scale questions due to their cultural level or knowledge of the Spanish language; or patients receiving drug treatment likely to interfere with their ability to understand or answer the scale questions.

Sample size was estimated with respect to sensitivity of the GAD-7 scale for diagnosing the target disease (GAD). One hundred participants with GAD were required to ensure that the total width of the 95% CI around a sensitivity proportion of 0.90 was no greater that 0.05, assuming an estimated prevalence of GAD in clinical populations in Spain ranging from 6% and 8% [1,8,13]. A similar sized age- and sex-matched control group was also enrolled. Sample was increased by 5% to prevent information losses in the statistical analysis. A total sample size of 210 subjects was therefore considered adequate.

The retest sample was selected at random as a sub sample from the scaling and validation sample. Clinicians were instructed to repeat the required measures in the first 2 out of each 10 patients enrolled by her or him.

### Concurrent validity

The following scales were used to assess the concurrent validity of the adapted version of the GAD-7 scale:

#### Hamilton Anxiety Scale (HAM) [14]

This is a 14-item, clinician-rated scale formulated as a semi-structured interview to assess the anxiety level of the subject. Items are scored from 0 (absent) to 3 (severe). The total score ranges from 0 and 42 points and may be categorized into four severity groups: normal (0-9), mild (10-15), moderate (16-24), and severe (25-42).

#### Hospital Anxiety and Depression Scale (HADS) [15]

This is a 14-item, self-administered scale in which anxiety and depression are assessed by 7 items each. Each item is scored from 0 to 3 with several anchors. Some items are assessed positively and others negatively. A score ranging from 0 and 21 points may be obtained in each domain. The score in each domain may be categorized into four severity groups: normal (0-7), mild (8-10), moderate (15-21) and severe (15-21).

#### World Health's Organization Disability Assessment Scale (WHO-DAS II 12-item version) [27,28]

This is a 12-item, self-administered scale. Items are grouped by pairs in 6 domains: 1-Understanding and communicating with the world, 2-Moving and getting around, 3-Self care, 4-Getting along with people, 5-Daily life activities (household responsibilities, leisure, and work), and 6-Participation in society. This scale contains 5 additional items, one about overall health and four about the number of days with activity limitations in daily life. Scoring is standardized on a 0-100 metric scale, where 0 means no disability and 100 the highest disability.

### Statistical analysis and psychometric studies

A descriptive analysis including measures of central tendency and dispersion and Gaussian curve fitting using a Kolmogorov-Smirnov test was first performed. The study of the psychometric properties of the GAD-7 scale focused on three aspects: feasibility, reliability, and validity. *Feasibility: *this section recorded the time taken by patients to complete the scale, the difficulties encountered by patients for answering questions, and the number of missing values (non-answering patients) for each question. Items were analyzed by calculating the frequency of each response category within each item, as well as the blank response rate for each item. Floor and ceiling effects were also analyzed, both in each item and in the overall questionnaire. *Reliability: *reliability was assessed by Cronbach's alpha calculation [29] for internal consistency, and by test-retest stability estimation using the correlation between presentations and the intra-class correlation coefficient. *Validity: *(a) *Construct*: structural validity (or construct validity) was assessed by exploratory factor analysis [30] using the common factor model, the principal component extraction method, and Oblimin rotation (if rotation was possible). The K1 rule (eigenvalues greater than one) and Cattell's screen test were used to decide on the number of factors contained in the solution. A confirmatory factor analysis [31,32] was also performed to corroborate the original structure, assuming the existence of one single factor where all items loaded. The maximum likelihood estimation method was used by means of LISREL 8.8 software. (b) *Discriminant validity: *discriminant validity was calculated by dividing the sample into quartiles based on the GAD-7 total score and comparing the upper to the lower quartile, both for the mean scores in individual items and for the overall score. For other clinical criteria, validity was assessed by calculating the mean-related difference between the GAD-7 mean overall score of the diagnostic groups assigned by the clinical investigator, A Student's t-test, Mann-Whitney's U test for independent groups, or one-way ANOVA were used, depending on the number of groups compared. (c) *Criterion validity: *diagnostic performance curves (ROC curves) were analyzed, and sensitivity and specificity rates, as well as the positive and negative predictive values of the questionnaire, were calculated when the resulting diagnostic classification was compared to the clinical diagnosis of reference. (d) *Convergent validity: *The degree of concordance between the GAD-7 scale and the Hamilton Anxiety Scale (HAM-A), the Hospital Anxiety and Depression Scale (HADS, anxiety domain), and the WHO-DAS II disability questionnaire was calculated. Concordance between classification criteria was assessed by inter-rater agreement statistics (kappa) and a Chi-square test.

All statistical calculations were performed using SPSS for Windows v15.0 routines.

## Results

The pilot sample included 62.5% of males with a mean age of 50.38 years (SD = 16.76 years, min = 32, max = 83). Among these, 28.6% had primary education, 42.9% secondary education, 14.3% higher education or vocational training, and 14.3% had finished a degree. Mean questionnaire completion time was 2 minutes and 30 seconds (SD = 0:01:24), with values ranging from 5 minutes in one subject and 30 seconds in two subjects. No items in the questionnaire were left blank, and no response accumulation at the ends of the scale was observed. No patient diagnosed with GAD asked for any help from the clinician in charge or needed any clarification. In the control group, an 83-year-old woman reported that she needed help to answer the questionnaire because she did not know where to mark the answers. Three control subjects reported difficulty in answering some of the questions, particularly question 5. Only the 83-year-old woman reported having asked for clarifications. An initial check in the pilot sample of item discrimination ability between patients and controls showed that all items were able to detect significant differences between groups (p < 0.05), except for item 5 (p = 0.105).

A total of 212 patients with full information were recruited for the validation and scaling sample. Mean patient age was 47.59 years (SD = 15.8), with values ranging from 19 and 85 years, and 72.6% of patients were female. Mean body mass index (BMI) of sample patients was 25.97 (SD = 4.5) kg/m^2^, with values ranging from 16.53 and 45.7. Ninety-nine percent of patients were Caucasian and only 1% were black. Four percent had not finished elementary school, while 33% had finished elementary school, 27% secondary school, 15% vocational training, and 21% higher education. As regards marital status, 17% were single, 66% married, 8% divorced or separated, 8% widowed, and 1% other. As to occupational status, 20% of patients were housewives, 61% were working, 4% unemployed, 6% occupationally disabled, and 9% retired.

According to the initial sample design, 50% of patients belonged to the group diagnosed with GAD and 50% to the control group. Sex- and age-matching of both sub samples was verified. The difference between mean ages in both groups was 0.9 years (SE = 2.18), which was considered non-significant (p = 0.679). No significant differences were also found in all other sociodemographic variables: sex (p = 1.00); race (p = 0.161); level of education (p = 0.262); marital status (p = 0.596); occupational status (p = 0.095).

As expected, a significant difference was found between the clinical and control groups in ongoing treatment. Seventy-nine percent of patients in the GAD group and only 20% in the control group were receiving any treatment (p < 0.001). Patients in the GAD group had a maximum of 6 treatments, although most patients had 1 or 2 treatments. Twenty-nine GAD patients were receiving no treatment. In the group diagnosed GAD, the duration of disorder ranged from 0 and 22 years, with a mean of 3 and a half years (SD = 3.9). The most commonly used treatments included SSRI and SNRI antidepressants (44%), followed by long-term (34%) or short-term (28%) benzodiazepines.

### Feasibility

All subjects answered all questions in the GAD-7 questionnaire, i.e. no blanks were recorded. Hence, there seems to be neither item comprehension problems nor likelihood of inadequate choice of the terms used as response scale anchor points in the adapted questionnaire. Completion time was short (around 2 and half minutes). Overall, answers were suitably distributed throughout all answer categories. There was a slight tendency to a floor effect in questions 4 (worried about different things) and 7 (afraid as if something awful might happen), with 42% and 51% accumulation of answers in the lowest rating ("not at all") respectively. Because scale assessment is performed with both GAD and control subjects, these two items may be considered as particularly "difficult" to comply with in the absence of GAD. All items showed some degree of positive asymmetry, with a tendency for answers to accumulate in the lowest rating categories; however, answer distribution in items 1 through 4 may be considered symmetric.

### Dimensionality and scaling

In the exploratory factor analysis, only an eigenvalue greater than one was obtained, which would account for the 72% variance found in the correlation matrix. All items had positive loadings higher than 0.75 in the single domain (Table 1), and the proportion of explained variance from each individual item (communality) ranged from 0.576 (item 6) and 0.798 (items 1 and 3). Since only one domain was obtained, the solution was not rotated.

**Table 1:** GAD-7 scale item loading in the 1 component exploratory factor solution and explained variance.

Item	Component
Nervous	0.893
Stop worrying	0.880
Excessively worried	0.893
Restless	0.870
Difficulty in relaxing	0.840
Easily irritated	0.757
Afraid of something awful	0.811

Explained variance	72,341

**Figure 1:**
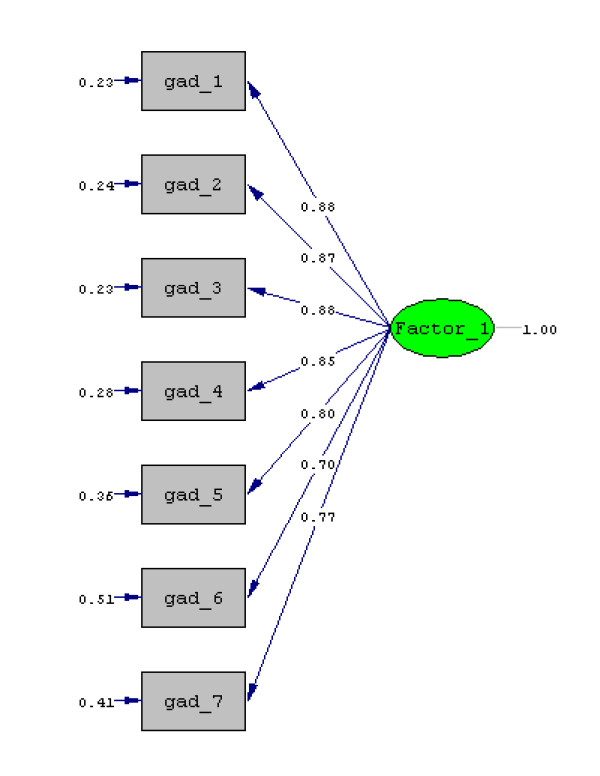
**Maximum likelihood confirmatory solution of items in the adapted version of the GAD-7 scale. Standardized loadings**. Chi-Square = 32.59, df = 14, p-value = 0.00330, RMSEA = 0.080

The confirmatory factor solution obtained (Figure 1) produced a good fit, with a Chi-square/df ratio value of 2.8, i.e. lower than 3, as recommended [33]. GFI statistics reached a value of 0.96 (> 0.95), AGFI = 0.91 (> 0.90), CFI = 0.99 (> 0.90), NFI = 0.98 (> 0.90), and RMSEA = 0.080 (≤0.080). All estimated loadings were significant (p < 0.001), the sign obtained was as expected, and the model was correctly identified.

### Reliability

Cronbach's alpha reached an excellent value (0.936). All items showed a high item-total correlation (higher than 0.68), with a test-retest correlation of 0.844 and an intra-class correlation coefficient of 0.926 (95% confidence interval of 0.881-0.958). Comparative values obtained with HADS yielded a Cronbach's alpha at baseline of 0.820 (considered to be a good value), with a test-retest correlation of 0.938 and an intra-class correlation coefficient of 0.926 (95% confidence interval of 0.881-0.959).

### Inter-rater validity

All items reached the highest score for the congruence index in the GAD objective domain, with scores ranging from 0.50 (item 7) and the maximum possible value of 1 (item 5). As expected, negative index scores were found in all cases in the depression domain, with values ranging from -0.31 (item 7) and -0.75 (item 5). These results suggest that all items adequately measure the GAD concept. Although all congruence scores in the depression distractor domain were negative, the fact that three of the scores were somewhat distant from -1 (within -0.31 and -0.38) suggests that raters think that the corresponding items might also indicate the presence of some degree of depression, but always tangentially, since all items reach the maximum possible rating in the anxiety domain.

### Discriminant validity

The scores obtained with the selected sample covered the whole possible metric space, and scores of both 0 and 21 points were recorded. Mean score was 8.75 points, with a standard deviation of 6.44 points. The 95% confidence interval for the mean ranged from 7.88 and 9.62. Skewness statistics did not suggest lack of symmetry (skewness = 0.167; SE = 0.167), but visual inspection of score distribution suggested a slight positive bias, thus ruling out a normal distribution (K-S = 0.144; DF = 212; p < 0.001). This lack of normality may be attributed to the existence of two scoring subgroups (normal subjects and GAD subjects), assumed to originate from different reference populations.

When quartiles 1 and 4 were compared, all items yielded significant differences (p < 0.001) in all cases; variance was also different between the two groups in all items (p < 0.08). it may thus be concluded that each and every item is able to discriminate between high and low scores, and contributes to them.

The total GAD-7 score detected significant differences between the means recorded for the group diagnosed GAD (mean = 13.96, SD = 4.19) and the control group (mean = 3.54, SD = 3.32). The difference between the groups was significant (p < 0.001) and a trend towards different group variances was also suggested (p = 0.070).

### Criterion validity

**Figure 2:**
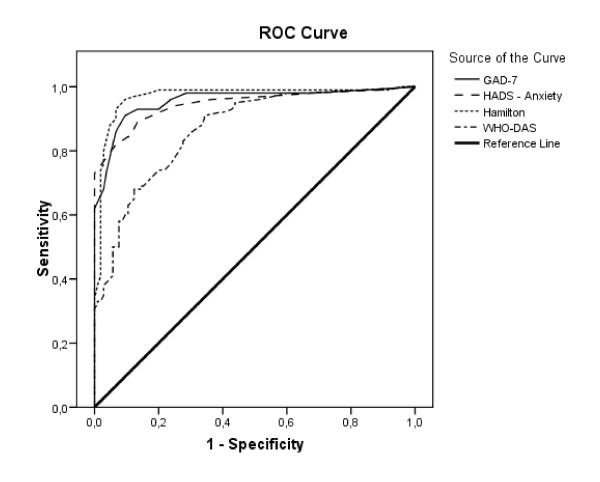
**ROC curve of GAD-7 total score**.

Since the area under the receiver operating characteristic curve (ROC curve) reached a value of 0.957 (SE = 0.014, p < 0.001), the null hypothesis of the area having a value of 0.5 in the population (and thus indicating the absence of inter-group discrimination) can be rejected. The 95% confidence interval ranged from 0.930 and 0.985, thus suggesting excellent discrimination. Use of a score of 10 as cut-off point resulted in a sensitivity of 86.8% and a specificity of 93.4%. Positive and negative predictive values were 92.9% and 87.6% respectively (Table 2). Figure 2 shows the ROC curves for the scores of GAD-7 and the concurrent measures. Areas under the curve with 95% confidence intervals for each individual measure follow. HADS-Anxiety: 0.942 (0.911-0.974), Hamilton: 0.970 (0.945-0.944), and WHO-DAS: 0.868 (0.820-0.916). Confidence intervals for GAD-7, HADS, and Hamilton overlapped, and should therefore not be considered statistically different. The WHO-DAS II scale showed a comparatively worse discriminant behavior.

**Table 2:** Operating characteristics of GAD-7 for different cut-off points.

Cut-off point	Sensitivity	Specificity	PPV	NPV	Cases
8	93.4	85.8	88.6	92.9	53.8
9	91.5	90.6	90.7	91.4	50.5
10	86.8	93.4	92.9	87.6	46.7
11	79.2	95.3	94.4	82.1	42.0
12	74.5	96.2	95.2	79.1	39.2
13	67.9	97.2	96.0	75.2	35.4
14	62.3	100.0	100.0	72.6	31.1

Note: PPV = Positive predictive value; NPV = Negative predictive value. All values expressed as %.

The score obtained in GAD-7 significantly correlated with the other questionnaires used in the study as concurrent measures (Table 3). A very high correlation was seen between GAD-7 and the HADS Anxiety scale (r = 0.903), and a high correlation was also seen with the depression scale (r = 0.713). Since a possible overlap of the depression and anxiety subscales and GAD-7 was suspected, an exploratory factor analysis was conducted with all items of both scales, which resulted in two differentiated factors: one containing all GAD-7 items plus items in the HADS Anxiety subscale, and the other containing the items in the HADS Depression subscale.

**Table 3:** Correlation of GAD-7 with other concurrent questionnaires used in the cultural adaptation study of the scale.

	GAD - Total score	Anxiety (HADS)	Depression (HADS)	Hamilton-A
Anxiety (HADS)	.903**	-	-	-
Depression (HADS)	.713**	.733**	-	-
Hamilton (A)	.852**	.835**	.760**	-
WHO-DAS Total score	.704**	.753**	.813**	.755**

** p < 0.001

The comparison of the GAD-7 scores of the four severity groups of anxiety diagnosed by the HADS-A yielded significant differences between the mean group scores (F = 205.3; DF1 = 3; DF2 = 208; p < 0.001). Different variances among groups were also seen (F = 3.64; DF1 = 3; DF2 = 208; p = 0.014). All severity groups had significantly different (p < 0.001) mean GAD-7 scores. Furthermore, there was agreement between the classification groups generated by both scales (Kappa = 0.493; p < 0.001).

**Figure 3:**
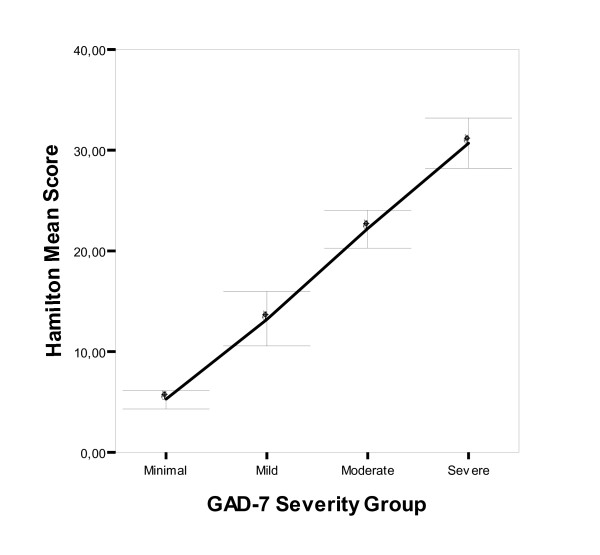
**Mean and confidence interval (95%) of HAM-A score by severity group according to the GAD-7 scale**. Anxiety group according to GAD-7 score; Normal/Minimum (0-7 points), Mild (8-10), Moderate (11-14) and Severe (15-21). p < 0.001 for between-group comparisons in all cases.  Diagonal segments are produced by ties.

A high correlation (r = 0.852) was also found between GAD-7 and the Hamilton Anxiety Scale (HAM-A) (Table 3). Differences were seen between the classification groups based on HAM-A in the mean values in the GAD-7 scale (F = 175.3; DF1 = 3; DF2 = 208; p < 0.001) and variances (F = 3.24; DF1 = 3; DF2 = 208; p = 0.023) (Figure 3). Multiple intergroup comparisons showed significant differences between all severity groups (p < 0.007). There was agreement between the classification groups generated by both scales (Kappa = 0.502; p < 0.001).

**Figure 4:**
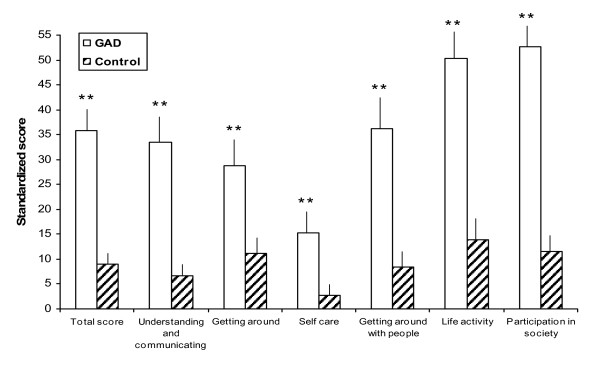
**Mean (95% CI) standardized score of WHO DAS II scale, total and by domain, by group of subjects**. GAD = Generalized Anxiety Disorder group; Control = Control group; **p < 0.001.

GAD-7 correlation with the WHO's Disability Assessment Scale was high and positive (Table 4), although somewhat lower (r = 0.704) than other concurrent correlations. Significant between-group differences were found (p < 0.001) when the total DAS scores of the control and GAD groups were compared. Significant differences were also found for all 6 DAS domains; higher disability scores were recorded in the group diagnosed GAD (Figure 4). While the GAD-7 scores showed different degrees of correlation with the different DAS domains (Table 4), all of them were significant (p < 0.001). The highest correlations were observed in the Participation in society (r = 0.741), Understanding and communicating with the world (r = 0.679), and Life activities (r = 0.638) domains, while the lowest ones were found in the Self care (r = 0.412) and Moving and getting around (r = 0.438) domains. GAD-7 scores significantly correlated (p < 0.001) with the number of days with activity limitations in daily life (r = 0.686) and with the number of days in which the patient was unable to carry out his/her tasks (r = 0.454). Similarly, significant albeit moderate correlations were found in the number of times patients has attended medical visits (either to primary care or specialist physicians) in the previous 4 weeks (Table 4).

**Table 4:** Correlation of GAD-7 with scores (total and by domain) in the WHO-DAS II disability assessment scale and with the number of medical visits (to primary care and specialist physicians) in the past 4 weeks.

WHO-DAS II	GAD-7
Total score	.696**
Understanding and communicating with the world	.679**
Moving and getting around	.438**
Self care	.412**
Getting along with people	.585**
Daily life activities	.638**
Participation in society	.741**
Medical visits	GAD-7
Primary care	.393**
Specialist	.373**

** p < 0.001

## Discussion

The version adapted into Spanish of the GAD-7 scale has a one-dimensional structure that matches the original structure based on DSM-IV diagnostic criteria. All items measure the same concept and in the same direction. Excellent reliability values were found both for homogeneity in concept measurement and stability between measurements when no clinical intervention was involved. The expert panel agreed that the construct assessed by the questionnaire was eminently one-dimensional and distinguishable from depression-related symptoms. In addition, the scores for the scale correlated well with those of other scales assessing anxiety, particularly the rater-administered HAM-A and the self-administered HADS. It must be taken into account that GAD patients also have symptoms of depression, although both conditions are discernible.

Each individual item in the scale, as well as the total score, adequately discriminated between control subjects and patients diagnosed GAD according to standard clinical criteria. Since a cut-off point of 10 yields excellent sensitivity and specificity values, and is in line with the cut-off value proposed by the original authors of the scale [22], this value is proposed for potential GAD diagnosis in standard clinical practice.

As noted by Spitzer et al. during the development of the original instrument [22], a relationship was also found between GAD assessments using the scale and the disability level assessed by the WHO-DAS II scale in several domains of daily life. This agrees with previous observations relating GAD to a high disability level in terms of work and daily life activities [5,34,35]. In addition, the group diagnosed GAD had the greatest number of ongoing treatments and a greater number of disability days experienced.

The GAD-7 scale adapted into Spanish has been shown to correlate well with two scales widely used to assess severity of anxiety symptoms in our setting and in other clinical environments. These scales, i.e. HAM-A administered by a trained interviewer or HADS self-administered by the patients, are used in daily clinical practice as GAD diagnostic support to assess symptom severity and to monitor the impact of healthcare interventions on this disorder. While not properly analyzed in this cultural adaptation study, future uses of the GAD-7 scale similar to those of HAM-A and HADS should not be dismissed. Indeed, the good performance of the new scale and its short administration time should be advantageous and provide for highly cost-effective administration. In fact, the cut-off points set to separate categories of symptom severity fully agree with those set for the HAM-A and HADS scales. It should be kept in mind that the original purpose of the GAD-7 scale was to screen patients suffering from this condition. While psychometric scaling properties and validity measures are promising, additional evidence will be needed in order to fully enable its use as a patient reported outcome measure. A literature review found no information about the meaningful clinical difference or the change expected in scores in follow-up studies.

While our adaptation study may have limitations, some of these should be attributed to the scale itself, rather than to the cultural adaptation study. Thus, in line with the comments issued during the scale pilot test, the questionnaire correction manual should include a warning on its use in elderly populations, emphasizing the possible need for clarification support or help when questions are read by patients, particularly very old patients. Because our study was conducted in medical centers representative of three autonomous communities only, its generalization to other cultural environments might be questioned. However, we think that the participating centers from Madrid, Barcelona, and Saragossa may be representative of the linguistic variability existing in the country, and may therefore be sufficiently representative of the whole national territory. However, use of this scale in Spanish-speaking environments culturally different from Spain should be tested in advance using appropriate psychometric studies.

In conclusion, the version adapted into Spanish showed excellent metric properties, and its high discriminant ability, briefness, and fast administration allow for its use in standard clinical practice for patient screening purposes. The ability of the scale to measure changes in patient conditions over time, or in response to treatment, remains to be elucidated. We hope that availability of this questionnaire to Spanish-speaking healthcare professionals will allow for increasing early detection and treatment of these patients, thus improving their quality of life and reducing medical and psychiatric complications.

## List of abbreviations

AGFI: Adjusted Goodness of Fit Index; ANOVA: Analysis of Variance; ASQ: Anxiety Screening Questionnaire; AUC: Area Under the Curve; BMI: Body Mass Index; CAS: Clinical Anxiety Scale; CFI: Comparative Fit Index; CRF: Case Report Form; DF: Degrees of Freedom; DSM: Diagnostic and Statistical Manual of Mental Disorders; ESEMeD: European Study of the Epidemiology of Mental Disorders; F: F statistic; GAD: Generalized Anxiety Disorder; GFI: Goodness of Fit Index; HADS: Hospital Anxiety and Depression Scale; HAM-A: Hamilton Anxiety scale; CI: Confidence Interval; ICD: International Code of Diseases; KS: Kolmogorov-Smirnov; NFI: Normed Fit Index; p: p value = Significance Level; RMSEA: Root Mean Square Error of Approximation; ROC: Receiver Operating Characteristic; SD: Standard Deviation; SE: Standard Error; SNRI: Selective Noradrenalin Reuptake Inhibitor; SSRI: Selective Serotonin Reuptake Inhibitor; STAI: State-Trait Anxiety Inventory; WHO-DAS II: World Health Organization Disability Assessment Scale II.

## Competing interests

Javier Rejas and Olga Freire are full-time employees of Pfizer, the company sponsoring this study. All other authors have no competing interests.

## Authors' contributions

The authors of this manuscript state that all of them have contributed substantially to manuscript preparation, interpretation of results or study design and logistics. JGC, EZ, MR, AP and JR were responsible for study design. MR and AP carried out data analysis and interpretation. MP and VLG participated in linguistic validation process and interpretation of data. OF was responsible for the logistics and conduct of the study. All authors participated read and approved the final manuscript.
